# Associations of bee sting injuries with environmental and social factors: an exploratory study

**DOI:** 10.3389/fpubh.2026.1742966

**Published:** 2026-02-27

**Authors:** Yoonhee Kim, Kyung-Duk Min

**Affiliations:** College of Veterinary Medicine, Chungbuk National University, Cheongju, Republic of Korea

**Keywords:** bee sting injuries, Moran's *I*, ordinary zero-inflated negative binomial, statistical models, veterinary epidemiology

## Abstract

**Introduction:**

Bee stings are a significant public health concern, yet their spatial and temporal patterns have been rarely examined. Because such analyses are important for developing control strategies, this study investigated the associations between bee sting injuries and various environmental and social factors in South Korea.

**Methods:**

An ecological study was conducted at the administrative district level, encompassing 250 districts across South Korea from 2014 to 2021. Annual counts of emergency department visits for bee sting injuries were analyzed in relation to meteorological, environmental, and demographic variables using a zero-inflated negative binomial (ZINB) regression model to account for excess zeros and overdispersion.

**Results:**

Significant positive associations were observed between bee sting injuries and relative humidity [relative risk (RR) = 1.012, 95% confidence interval (CI): 1.005–1.019] as well as forest coverage (RR = 1.024, 95% CI: 1.017–1.032) and deforested area (RR = 1.092, 95% CI: 1.028–1.160). Negative associations were found with temperature (RR = 0.982, 95% CI: 0.975–0.989) and urbanization (RR = 0.974, 95% CI: 0.969–0.980). The incidence of bee sting injuries showed a gradual decline over the study period, with an RR of 0.941 (95% CI: 0.910–0.974) per year.

**Discussion:**

Bee sting injuries are influenced by multiple environmental and social factors. Further research is warranted to confirm causal relationships and to guide evidence-based preventive strategies.

## 1 Introduction

Bee sting injuries can lead to serious medical complications such as anaphylaxis, respiratory distress, and cardiac arrest, particularly among vulnerable populations, including older adults and rural residents (1, 2). They also represent a significant public health concern. In Europe, annual mortality from hornet, wasp, and bee stings has been reported to range between 0.03 and 0.48 deaths per million people, amounting to hundreds of fatalities each year (3, 4). In South Korea, Health Insurance Review and Assessment Service (HIRAS) records from 2010 to 2014 documented 78,860 injuries and 49 deaths caused by Hymenoptera stings, corresponding to an annual average of 15,772 injuries and 9.8 fatalities. The total medical costs associated with wasp stings during this period were estimated at approximately 3.2 million USD (5).

It is important to investigate the environmental and social drivers of bee sting incidents to develop effective control strategies. Previous studies have shown that recognizing such factors can inform the creation of public health policies. For instance, analyses of climatic and ecological factors related to malaria transmission have supported the implementation of vector control programs, while identifying socioeconomic inequalities in injury occurrence has guided resource allocation for prevention strategies (6). In other words, clarifying the environmental and social factors associated with bee sting injuries is essential for designing targeted interventions and evidence-based policies to reduce their public health impact.

Several factors can be regarded as potential drivers of bee stings because of their plausible underlying mechanisms. First, temperature and sunlight levels may influence bee physiology, foraging behavior, and defensive responses, making them important candidates for epidemiological studies. Elevated temperatures are generally associated with increased bee activity and extended foraging periods, which may heighten the likelihood of human–bee encounters (7). Similarly, prolonged sunlight exposure throughout the year may extend diurnal foraging windows, thereby increasing the chances of spatial overlap between bees and human populations. Excessive heat or high solar radiation can also compromise colony health and reduce floral resources, which may indirectly increase human–bee interactions as bees expand their foraging ranges into anthropogenic environments (8–10). Second, precipitation and relative humidity may also play important roles. While increased precipitation can temporarily suppress bee activity by reducing flight opportunities, it may enhance floral abundance over time, indirectly supporting larger bee populations (11, 12). High humidity levels may further benefit nest maintenance by preserving adequate moisture within hive structures (13–15). Third, the proportion of protected areas, forest cover, and deforested land reflects habitat integrity and ecological disturbance. Extensive forest and protected regions provide stable nesting habitats, sustain floral and nesting resources, and may reduce colony displacement toward human settlements (16–18). By contrast, deforestation disrupts established nesting grounds, diminishes resource availability, and may encourage migratory behavior, thereby increasing the likelihood of human contact (19–23). Fourth, urban and agricultural land use patterns represent the intensity of human activity and may be associated with bee sting occurrence. Urban areas combine high human density, ornamental vegetation, and numerous structural nesting sites, all of which can increase the frequency of encounters between humans and bees (24–27). Agricultural regions, meanwhile, are characterized by heightened sting risk among outdoor laborers due to dense flowering crops and seasonal work patterns (12, 28, 29). However, agricultural activities may also negatively affect bee colonies through pesticide and insecticide use, as well as frequent human disturbance (30, 31).

In this study, we examined the epidemiological associations between potential environmental and social factors and bee sting injuries in South Korea. The outcome distribution showed excess zeros and overdispersion, and an ordinary zero-inflated negative binomial (ZINB) model was used to jointly model the count and zero components. Although the mechanisms underlying these factors have been discussed in previous research, epidemiological studies explicitly analyzing them remain limited. South Korea provides an especially suitable setting for this investigation because it offers systematic, region-specific data on bee sting injuries along with reliable, validated datasets for environmental and social variables from multiple sources. Bee sting injuries can be situated within a broader category of animal-related injuries associated with environmental change and land-use patterns. Climate variability and land-use modification have been reported to alter animal habitats and contact opportunities with human populations (32). In this context, bee sting injuries provide a specific and observable outcome for examining population-level associations with environmental and social factors.

## 2 Materials and methods

### 2.1 Study design

This ecological study was designed to examine the epidemiological associations between bee sting injuries and environmental as well as social factors across districts in South Korea from 2014 to 2021. Data on bee sting injury cases were collected alongside meteorological, demographic, and land-use variables—including temperature, relative humidity, precipitation, sunlight duration, population size, proportions of urban and agricultural land, and proportions of protected, forested, and deforested areas. These variables were compiled for 250 administrative districts (cities, counties, and districts) over 8 years, with the study unit defined as district by year (250 × 8). This study was reviewed and approved by the Institutional Review Board of Chungbuk National University (Approval No. CBIRB-202311-HR-024).

### 2.2 Outcome variable

The outcome variable was the annual count of bee sting injuries aggregated at the administrative district level by year. These data were obtained from the National Emergency Department Information System (NEDIS), a nationwide surveillance platform managed by the Korea Disease Control and Prevention Agency (KDCA). The NEDIS compiles real-time reports from emergency medical facilities, including diagnostic classifications, patient demographics, and institutional locations (33). Bee sting injuries were identified using the International Classification of Diseases, 10th Revision (ICD-10) code “X23,” which denotes “Contact with hornets, wasps, and bees,” as defined by the World Health Organization (34). For each district, case counts were extracted based on the location of the reporting emergency department to approximate exposure location. All records were anonymized prior to acquisition, and no personally identifiable information was included.

### 2.3 Explanatory variables

The explanatory variables used in this analysis were selected to represent meteorological and environmental factors potentially associated with the occurrence of bee sting injuries. All variables were compiled annually for each of the 250 administrative districts across South Korea from 2014 to 2021. Table 1 presents an overview of the explanatory variables. Meteorological data—including annual temperature, relative humidity, precipitation, and sunlight duration—were obtained from the Automatic Synoptic Observation System (ASOS), operated by the Korea Meteorological Administration (35). Daily observations from ASOS stations were aggregated into monthly means and then averaged across each year to generate annual values. Environmental variables were constructed from several national and global datasets. Forest cover and deforestation rates were derived from the Global Forest Change dataset developed by Hansen et al. (36), which provides annual estimates of canopy cover and deforested areas at a 30-meter spatial resolution. For each year, the proportion of forested area within each administrative district was calculated using classified canopy data, and the deforestation rate was estimated as the proportion of land that experienced forest loss during the corresponding year. In this study, deforested region (%) indicates annual forest loss within each district, and forest region (%) indicates forest cover within each district based on canopy data. Urban and agricultural land cover proportions were obtained from NASA's MODIS MCD12Q1 land cover classification product (37). The level of ecological preservation was assessed using data from the Environmental Geographic Information System (EGIS), based on the designated proportion of protected areas in each district. In this system, Level I denotes regions with the highest ecological value and conservation priority (38). Population data were retrieved from the Korean Statistical Information Service (KOSIS) (39), which provides annual district-level demographic information based on administrative resident registration.

**Table 1:** Summary of data sources during collection period (January 2014–December 2021).

**Item**	**Data source**
Medical records	National Health Insurance Service (NHIS)
Population	Korean Statistical Information Service (KOSIS)
Land cover code	MCD12Q1, MODIS/Terra+Aqua Land Cover Type (NASA)
Weather data	Automatic Synoptic Observation System (ASOS)
Administrative district code	Administrative Standard Code Management System, Ministry of the Interior and Safety
Deforestation rate	High-resolution global maps of 21st-century forest cover change, *Science* 2013, Hansen et al.
Forest cover percentage	High-resolution global maps of 21st-century forest cover change, *Science* 2013, Hansen et al.
Proportion of urban land cover	NASA Moderate Resolution Imaging Spectroradiometer (MODIS)
Proportion of agricultural land cover	NASA Moderate Resolution Imaging Spectroradiometer (MODIS)
Level of ecological preservation	Environmental Geographic Information System (EGIS)

### 2.4 Statistical analysis

Descriptive statistics were calculated to summarize meteorological, environmental, and land-use variables across administrative districts. Explanatory variables were expressed as means with standard deviations. Districts were classified into two groups based on bee sting injuries during 2014–2021 (≥1 vs. 0), and descriptive comparisons are presented in Table 2, with group sizes reported. Differences between groups were assessed using independent *t*-tests, assuming approximate normality given the large sample size. Independent *t*-tests were used to compare environmental and land-use characteristics between district groups.

**Table 2:** Association of meteorological and environmental variables with bee sting injuries.

**Variables**	Regions		***p*-value (*t*-test)**
	**Bee sting injuries** + **(*****n*** = **224)**	**Bee sting injuries – (*****n*** = **26)**	
Temperature, °C	13.2 (1.05)	13.0 (0.959)	
Humidity, %	68.4 (3.36)	68.3 (3.44)	0.5089
Precipitation, mm	102 (27.4)	101 (26.0)	0.6895
Sunlight, h/year	195 (10.8)	195 (10.3)	0.9452
Protected area, %	13.3 (12.1)	15.9 (15.5)	
Deforested region, %	30.9 (23.3)	33.2 (23.9)	0.654
Forest region, %	0.0974 (0.135)	0.0942 (0.120)	0.0587
Agricultural land, %	14.5 (18.4)	13.8 (18.6)	0.4563
Urban region, %	28.8 (32.8)	25.8 (32.0)	0.0809

Data are presented as mean (standard deviation). ^***^p < 0.001.

The primary analysis employed an ordinary zero-inflated negative binomial (ZINB) regression to evaluate associations between explanatory variables and bee sting injury occurrence. This modeling approach was chosen due to the expected excess zeros and overdispersion observed in the outcome distribution, which made the Poisson model inappropriate. The ZINB model consists of two components: a negative binomial count model estimating relative risks (RRs) for districts reporting cases, and a logit model estimating odds ratios (ORs) for structural zeros. In the zero-inflation (logistic) component of the model, protected area (%), agricultural land (%), and urban region (%) were included as explanatory variables to represent districts in which the probability of reported bee sting injuries may be persistently low. These variables were selected to reflect long-term contextual characteristics related to exposure opportunity and reporting environment, thereby distinguishing structural zeros from zeros arising through stochastic variation in the count process (40–43). The model formula was expressed as:


$$\begin{array}{l} \text{Pr}\left({\text{y}}_{\text{i}}=\text{j}\right)=\left(1-{\text{π}}_{\text{i}}\right)\text{ g}\left({\text{y}}_{\text{i}}\right)\text{ if j}>0\;\left(\text{count}\right)\\ {\text{π}}_{\text{i}}+\left(1-{\text{π}}_{\text{i}}\right)\text{ g}\left({\text{y}}_{\text{i}}=0\right)\text{ if j}=0\;\left(\text{zero}\right) \end{array}$$

In the formulation, y_i_ is the observed number of bee sting injuries in district i. π_i_ is the probability that the observation is a structural zero. The count component included temperature (°C), humidity (%), precipitation (mm), sunlight (h/year), protected area (%), deforested region (%), forest region (%), agricultural land (%), and urban region (%). The zero-inflation (logistic part) component included protected area (%), agricultural land (%), and urban region (%). Year was modeled as a continuous variable centered at the first study year. Population size was included as an offset term [log(population)] in the count component. Annual district-level observations were analyzed using an ordinary ZINB model without district-level random effects. Multicollinearity was assessed using variance inflation factors (VIFs), and variables with a VIF of ≥10 were excluded. Model specification was guided by the distributional characteristics of the data, including the presence of excess zeros and overdispersion. AIC values for alternative count model specifications are provided in Supplementary Table S1. The distribution of bee sting injuries showed a substantial proportion of zero counts, with 469 zeros among 2,000 district–year observations (23.4%), indicating the presence of excess zeros. Overdispersion was assessed to evaluate the suitability of a Poisson model, and the results indicated that a Poisson specification was not appropriate. Given the presence of excess zeros and overdispersion, a zero-inflated negative binomial (ZINB) framework was applied for the analysis To assess potential spatial autocorrelation, Moran's *I* statistic was calculated prior to model estimation. Regression coefficients were exponentiated to obtain RRs and ORs, with 95% confidence intervals (CIs) calculated as ±1.96 times the standard error. After model estimation, residuals were mapped to administrative districts, and spatial autocorrelation was evaluated using Moran's *I* statistic under a Queen contiguity-based spatial weights matrix. A spatial ZINB model was considered as a potential extension, but was not fitted due to the absence of significant residual spatial autocorrelation.

Visualization of effect estimates was conducted using forest plots, and spatial patterns were mapped in R using the sf, ggplot2, and ggspatial packages. All analyses were performed using R version 4.2.2 (R Core Team, Vienna, Austria).

## 3 Results

**Figure 1:**
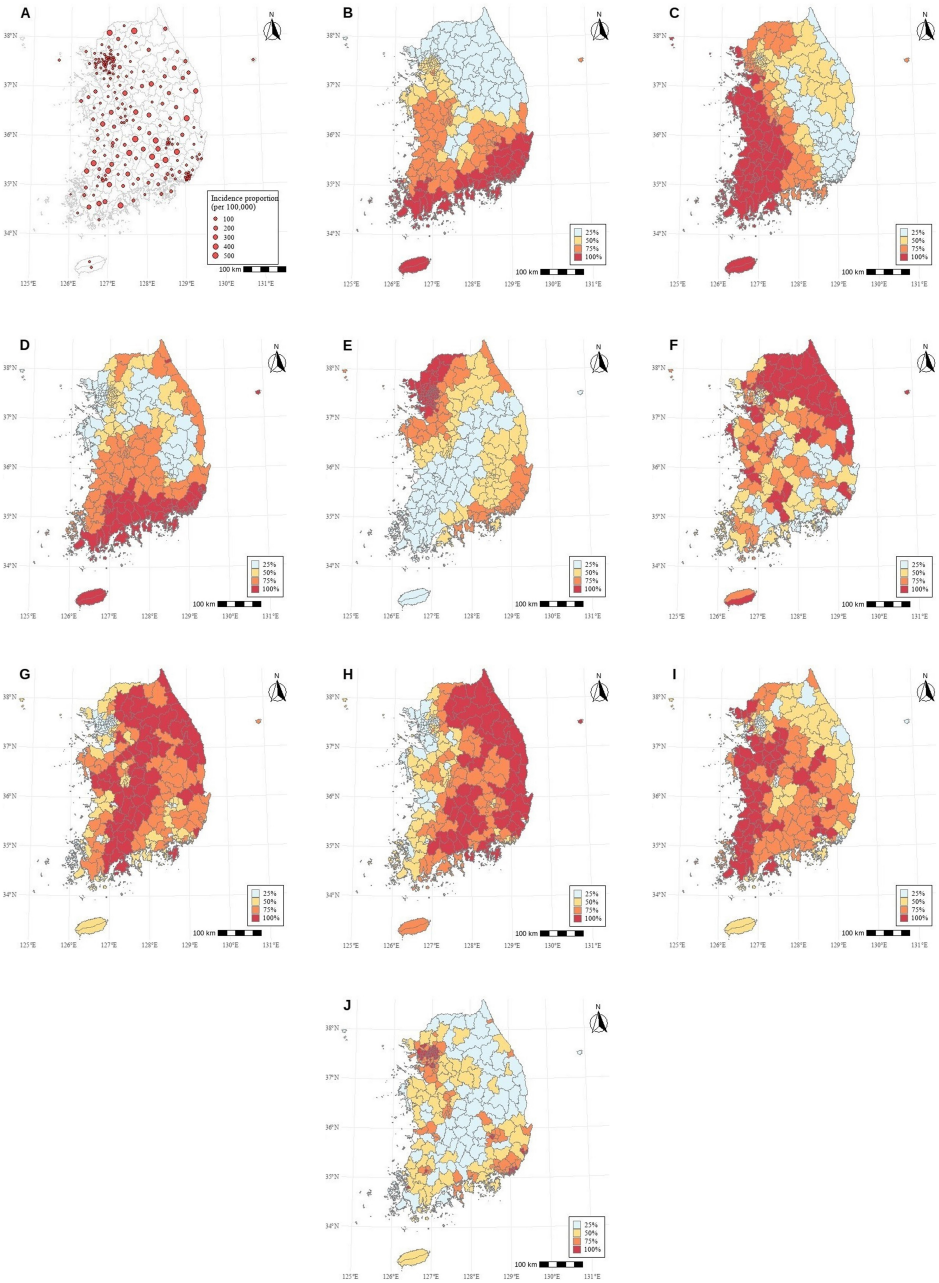
Incidence proportion of bee sting injuries by environmental variables (quartiles, 2014–2021). **(A)** Bee sting incidence proportion. **(B)** Annual temperature. **(C)** Annual humidity. **(D)** Annual precipitation. **(E)** Annual sunlight. **(F)** Proportion of protected area. **(G)** Deforested region proportion. **(H)** Forest region proportion. **(I)** Agricultural land proportion. **(J)** Urban region proportion.

In total, 2,000 observations (8 years × 250 regions) were included in the analysis. During the study period, 20,161 cases of bee sting injuries were reported in 2014, declining to 10,625 cases in 2020, with a subsequent increase to 12,762 cases in 2021. The observed annual number of bee sting injuries is presented in Supplementary Table S1. Annual counts of bee sting injuries ranged from fewer than 100 to more than 300 across districts. Group comparisons indicated that districts with bee sting injuries had a significantly higher mean annual temperature (13.2 °C vs. 13.0 °C, *p* = 0.0002) and a significantly lower proportion of protected area (13.3 vs. 15.9%, *p* = 0.0002) than districts without cases, while other variables showed no significant differences (Table 2). Spatial mapping of bee sting injury incidence proportion are shown in Figure 1A. Quartile maps of temperature, humidity, precipitation, sunlight, proportion of protected area, deforested region proportion, forest region proportion, agricultural land proportion, and urban region proportion are shown in Figures 1B–J.

**Figure 2:**
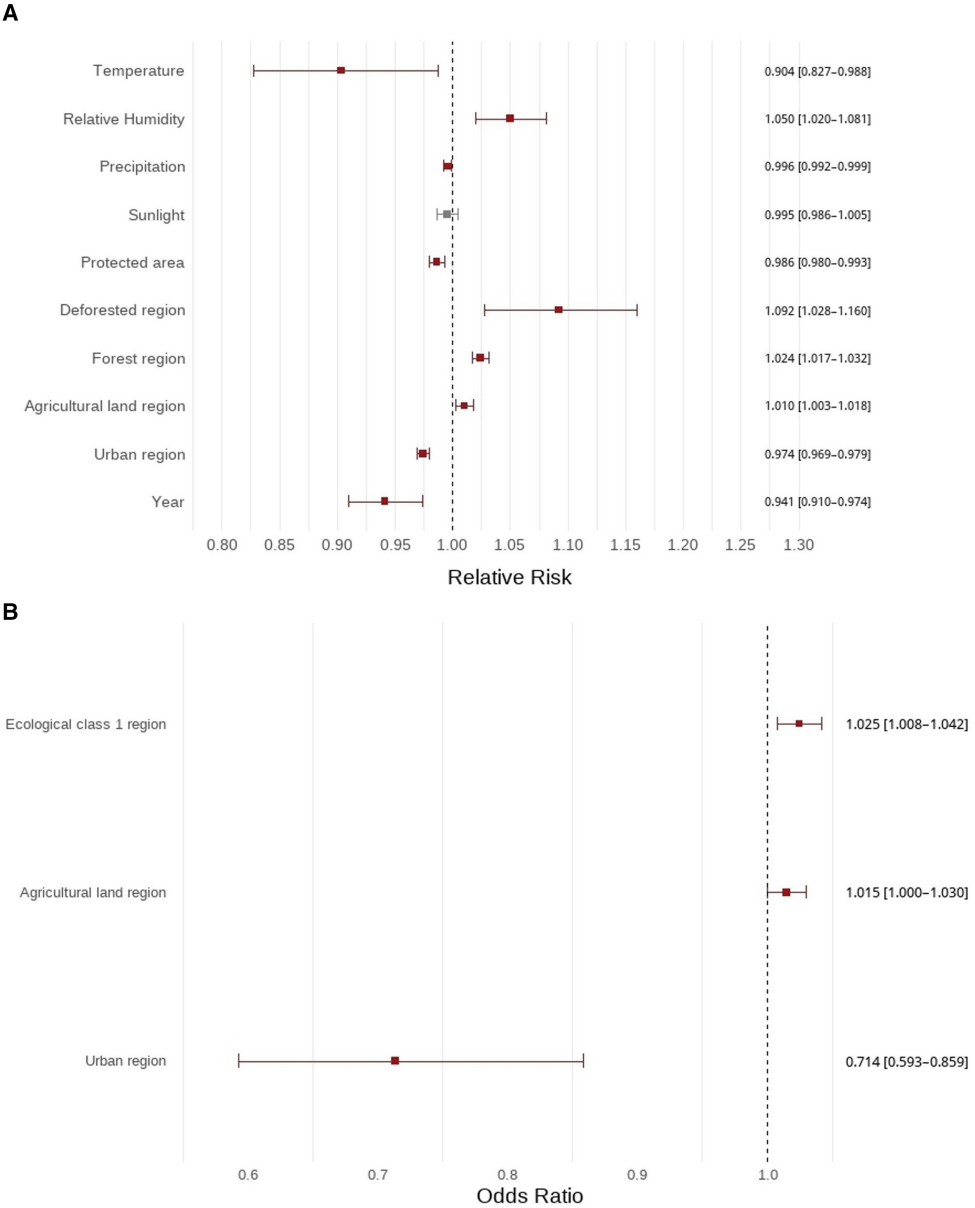
Forest plot of ordinary ZINB model results (count and zero components) for bee sting injuries. **(A)** ZINB count model forest plot. **(B)** ZINB zero model forest plot.

In the count component of the ordinary ZINB model, a 1% increase in forest coverage was associated with a 2.4% increase in bee sting injuries (RR = 1.024, 95% CI: 1.017–1.032). A 10% increase in deforested area corresponded to a 9.2% increase in injuries (RR = 1.092, 95% CI: 1.028–1.160). A higher urban proportion was inversely associated with bee sting injuries (RR = 0.974, 95% CI: 0.969–0.980), while annual precipitation also showed a negative association (RR = 0.996, 95% CI: 0.992–0.999). Annual humidity, agricultural land proportion, proportion of protected area, and annual temperature were also significantly associated with injury counts, whereas annual sunlight duration was not. In the zero-inflation component, agricultural land proportion (OR = 1.015, 95% CI: 1.000–1.030) and proportion of protected area (OR = 1.025, 95% CI: 1.008–1.042) increased the probability of observing zero cases, while a higher urban proportion decreased this probability (OR = 0.714, 95% CI: 0.593–0.859). Estimates of RRs and ORs with 95% CIs are presented in Figure 2. The Moran's *I* statistic for residuals from the ordinary ZINB model was 0.034 (*p* = 0.183), indicating no significant residual spatial autocorrelation. A spatial ZINB model was not applied because Moran's *I* for the ordinary ZINB residuals was not statistically significant. Temporal correlation was not modeled as a separate correlation structure. Year was included in the ordinary ZINB model to account for temporal variation.

## 4 Discussion

This study examined the environmental and social factors associated with bee sting injuries across 250 administrative districts in the Republic of Korea from 2014 to 2021. A gradual decline in incidence was observed over the study period. Among meteorological variables, mean temperature was negatively associated with bee sting injuries, whereas relative humidity was positively associated. Precipitation showed a negative association, and sunlight was not statistically significant. Regarding land-use factors, a higher proportion of deforested areas was positively associated with bee sting injuries, whereas urban region (%) showed a negative association in the count component of the ordinary ZINB model. In contrast, forest region (%) was positively associated with bee sting injuries. The proportion of agricultural land showed no significant association with injury occurrence. Overall, the findings suggest that environmental and social factors influence the spatial distribution of bee sting injuries, although some variables exhibited null associations.

Temperature was negatively associated with bee sting injuries in this study. A hospital-based analysis from Switzerland reported that the frequency and severity of Hymenoptera stings increased with higher daily temperatures, showing a marked rise when the mean temperature exceeded 24 °C (44). Similarly, population-level evidence from Alaska indicated that warmer summer periods were accompanied by a greater number of insect sting reactions requiring medical attention (45). These findings suggest that elevated temperatures are generally associated with an increased occurrence of sting-related injuries across diverse climatic regions.

Relative humidity showed a positive association with bee sting injuries in the present study. By contrast, hospital-based surveillance in Switzerland reported that lower humidity was associated with a higher frequency of bee sting injuries and more severe reactions (44). Meanwhile, reports from Northeastern Brazil documented seasonal clustering of bee sting injuries during warm and humid months, although humidity-specific estimates were not calculated (46). These findings indicate heterogeneity in the relationship across studies—an inverse pattern in a temperate European setting and higher occurrence in subtropical regions during humid periods. Direct epidemiologic evaluations of humidity in relation to bee sting injuries remain limited. Further investigations, including meta-analyses, may be warranted to clarify this association because such approaches allow the integration of heterogeneous findings and the assessment of consistency across studies.

Precipitation was negatively associated with bee sting injuries in this study, although epidemiological evidence on this relationship remains limited. In Switzerland, hospital-based surveillance of Hymenoptera stings reported that days with sting-related admissions had lower mean rainfall than non-sting days (*p* = 0.007) (44). Similarly, a Korean study of emergency calls for wasp stings identified an inverse association between daily rainfall and sting-related incidents, supporting the observation that increased precipitation suppresses sting occurrence (5). A study of European wasp ecology in metropolitan Adelaide also reported a weak negative correlation between total rainfall and nest destruction, though it did not examine human sting incident rates (47). Thus, consistency with the present findings is partial. Overall, the current results align with general seasonal patterns described in previous research, while differing in that precipitation was explicitly included as an explanatory factor in this analysis.

The proportion of protected areas was also associated with a higher occurrence of bee sting injuries in this study. In Sri Lanka's central hill country, sting injuries were reported within the Victoria–Randenigala–Rantembe Sanctuary, a designated forest reserve, with cases presenting to regional hospitals during periods of field activity in adjacent settlements (48). In the United States, surveillance of visitor injuries in Shenandoah National Park recorded cases of respiratory distress following insect stings or bites—including Hymenoptera stings—indicating that sting-related medical encounters can occur within large protected regions (49).

The proportion of deforested areas was positively associated with bee sting injuries in the present study. Previous epidemiological research has reported higher sting incidence in regions characterized by intensive human land use but has not directly assessed the proportion of deforested areas. In Ceará, Brazil, 1,307 cases were recorded between 2007 and 2013, with a predominance in urban regions (60.7 vs. 34.5%) (50). Similarly, in Campina Grande, Paraíba State, incidence was higher in urban than in rural zones, although deforested area was not evaluated as a specific variable (46). Thus, consistency with the present findings was partial—prior studies have noted elevated sting incidence in human-modified environments, but the proportion of deforested regions has rarely been explicitly examined.

Forest coverage was associated with a higher incidence of bee sting injuries in this study. In Sri Lanka's central hill country, a hospital-based analysis reported that most sting injuries occurred during outdoor activities in tea estates and settlements located within or near forest reserves, indicating frequent human exposure in forest-associated environments (48). Another study conducted in Anuradhapura District, Sri Lanka, found that 9.6% of all hospital admissions over 1 year were attributable to hornet stings, with most incidents occurring in paddy fields and chena cultivations situated near tropical dry evergreen forests (51). In Japan, an occupational epidemiological survey showed that more than 90% of forestry workers had experienced Hymenoptera stings, and systemic reactions were observed in 21% of them—a substantially higher proportion than among office-based controls (52). In Switzerland, a 5-year hospital-based analysis reported that 34% of patients presenting with Hymenoptera stings exhibited anaphylactic reactions, with a higher frequency of incidents in peri-urban and rural areas located near forested landscapes (44).

Bee sting injuries showed lower counts in districts with a higher proportion of urban areas according to the present findings. In Brazil, ecological studies reported that most bee sting injuries occurred in urban settings, suggesting greater exposure opportunities in densely populated regions (46). In Korea, severe systemic reactions following bee sting injuries were most often managed in metropolitan hospitals, reflecting the concentration of cases in large urban centers, despite the absence of spatially continuous urbanization indicators (53). Additionally, an analysis of emergency calls for wasp nest removal in Korea demonstrated a strong correlation with sting-related injuries in densely populated urban areas, further emphasizing the burden in metropolitan contexts (5). Although these studies differed in outcome definitions and exposure assessments, they consistently reported a high burden of bee sting injuries in urban environments. The consistency across different settings highlights the high burden of bee sting injuries in urban environments, although the present study showed a negative association between urban region (%) and injury counts in the count component of the ordinary ZINB model.

The proportion of agricultural land was not associated with bee sting injuries in this study. In Sri Lanka, hospital-based records from Anuradhapura District showed that a large proportion of sting injuries originated in paddy fields and chena cultivations within agricultural landscapes contiguous with forested regions (51). In Spain, a population survey in rural Mediterranean regions reported a higher prevalence of Hymenoptera sting reactions among agrarian populations compared with urban groups (54). Similarly, a population-based cohort study in Germany found increased Hymenoptera venom sensitization and systemic reactions among adults, with rural residence identified as a contributing factor (55). These findings contrast with the absence of a significant association in the present study and suggest heterogeneity across settings, likely reflecting differences in agricultural practices, exposure environments, and study designs.

These findings may be interpreted within the context of urbanization and land-use patterns characteristic of South Korea, where high-density residential development is closely interwoven with managed green spaces and peri-urban natural environments (56). Differences between highly urbanized districts with formalized environmental management and rural or forest-adjacent districts with comparatively limited protective infrastructure may be relevant to exposure conditions among districts reporting bee sting injuries (57). Furthermore, the concurrent associations observed for forest coverage and deforested areas may be considered in relation to landscape fragmentation in South Korea, where extensive forest cover coexists with localized land disturbance, rather than as opposing ecological processes (58).

This study has several limitations. First, the analysis was based on emergency department–derived surveillance data, which excluded cases treated in outpatient settings and likely led to an underestimation of bee sting injuries. Bee sting injury reporting during 2020 and 2021 may have been influenced by the COVID-19 pandemic and associated public health regulations, which could have affected healthcare-seeking behavior and outdoor activity patterns. As an ecological study, the present analysis is also subject to limitations in causal interpretation at the individual level, and residual confounding by unmeasured factors cannot be excluded (59, 60). Previous research has shown that incidence rates of psychiatric disorders were lower when outpatient data were omitted, suggesting a similar limitation for emergency department–restricted surveillance (61). Second, variations in access to emergency medical facilities—particularly in rural or remote regions—may have contributed to underreporting or misclassification. Evidence from healthcare access studies indicates that geographic barriers and limited regional connectivity are associated with reduced utilization of services in underserved areas (62). Third, unmeasured ecological and socioeconomic factors, such as floral resource availability, pesticide use, and microclimatic variability, were not incorporated. Methodological studies have demonstrated that residual and unmeasured confounding can account for variability in observed associations (63). Finally, the use of aggregated district-level data limited analyses to broad spatial scales and did not capture individual-level exposures, reducing the precision of the associations.

Despite these limitations, the results indicate that environmental, social, and land-use factors are associated with the occurrence of bee sting injuries and have important implications for prevention and planning. Regions characterized by higher urbanization or ongoing deforestation may require targeted awareness campaigns and strengthened emergency medical preparedness (64). The observed associations with temperature, humidity, and precipitation suggest that climate variability may influence future patterns of bee sting injuries (5). These findings underscore the importance of adaptive risk monitoring. Further research is warranted to incorporate outpatient and primary care data to improve case ascertainment, and to include additional variables such as floral abundance, pesticide exposure, and socioeconomic indicators, and to utilize spatially refined datasets for greater resolution (16, 65). Field-based investigations and multidisciplinary approaches—including behavioral and genetic studies of bee populations—are needed to provide a more comprehensive understanding of the ecological relationships underlying these patterns (66, 67). Such efforts are expected to support the development of region-specific strategies, enhance early warning systems, and inform policy measures addressing the public health burden of bee sting injuries under diverse environmental and social conditions.

## Data Availability

The original contributions presented in the study are included in the article/Supplementary material, further inquiries can be directed to the corresponding author.

## Supplementary material

Supplementary Figure S1 Annual number of observed bee sting injury cases in South Korea from 2014 to 2021. The bar chart illustrates temporal variation in total reported cases across study years, with a peak observed in 2016 and a decline in 2020 followed by a partial increase in 2021.
