# Supplementary figures and images for: Associations of bee sting injuries with environmental and social factors: an exploratory study
Source: Front Public Health. 2026 Feb 27;14:1742966. doi: 10.3389/fpubh.2026.1742966 (PMC12982371; doi:10.3389/fpubh.2026.1742966)

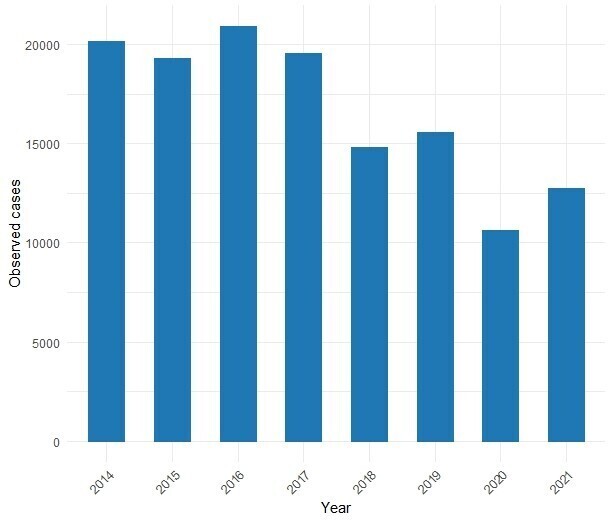

Supplement: Supplementary Figure S1 — Annual number of observed bee sting injury cases in South Korea from 2014 to 2021. The bar chart illustrates temporal variation in total reported cases across study years, with a peak observed in 2016 and a decline in 2020 followed by a partial increase in 2021. [file Image_1.jpeg]
