# Supplementary material for: Associations of bee sting injuries with environmental and social factors: an exploratory study
Source: Front Public Health. 2026 Feb 27;14:1742966. doi: 10.3389/fpubh.2026.1742966 (PMC12982371; doi:10.3389/fpubh.2026.1742966)
Supplement: Supplementary file 2 [file Table_1.docx]

**Supplementary Table S1:** Akaike Information Criterion (AIC) Values Comparisons for Candidate Models Evaluated for the Primary Analysis of Bee Sting Injuries.

| **Models** | **N_used** | **AIC** | **Delta_AIC** |
| --- | --- | --- | --- |
| Zero-inflated Negative Binomial model | 2,000 | 6986 | 0 |
| Negative Binomial model | 2,000 | 6993 | 7 |
| Zero-inflated Poisson model | 2,000 | 11643 | 4658 |
| Poisson model | 2,000 | 13817 | 6831 |

All models were fit on the same complete-case sample (N = 2000) using an identical covariate specification with offset(log(POP)), and the ZIP and ZINB models used the same zero-inflation covariates.
