# Supplementary material for: Associations of bee sting injuries with environmental and social factors: an exploratory study
Source: Front Public Health. 2026 Feb 27;14:1742966. doi: 10.3389/fpubh.2026.1742966 (PMC12982371; doi:10.3389/fpubh.2026.1742966)
Supplement: Supplementary file 3 [file Table_2.docx]

**Supplementary Table S2:** Variance Inflation Factors (VIFs) for Explanatory Variables used in the Ordinary Zero-Inflated Negative Binomial Model of Bee Sting Injuries.

| **Variable** | **N_used** | **VIF** | **Df** |
| --- | --- | --- | --- |
| Temperature | 2,000 | 2.189932 | 1 |
| Relative humidity | 2,000 | 2.366212 | 1 |
| Precipitation | 2,000 | 3.086356 | 1 |
| Sunlight | 2,000 | 3.956573 | 1 |
| Protected area | 2,000 | 1.275510 | 1 |
| Deforested region | 2,000 | 1.764907 | 1 |
| Forest region | 2,000 | 6.249498 | 1 |
| Agricultural land region | 2,000 | 3.230600 | 1 |
| Urban region | 2,000 | 5.705693 | 1 |
| Year | 2,000 | 6.342760 | 7 |

VIF values are presented for the variables assessed for multicollinearity. Variables with VIF ≥ 10 were excluded from model consideration.
